# Early effective treatment of small pulmonary nodules with video-assisted thoracoscopic surgery combined with CT-guided dual-barbed hookwire localization

**DOI:** 10.18632/oncotarget.17044

**Published:** 2017-04-11

**Authors:** Han Qi, Chao Wan, Liang Zhang, Junye Wang, Ze Song, Rong Zhang, Zhenfeng Zhang, Weijun Fan

**Affiliations:** ^1^ Department of Imaging and Interventional Radiology, Sun Yat-sen University Cancer Center, State Key Laboratory of Oncology in South China, Collaborative Innovation Center of Cancer Medicine, Guangzhou, China; ^2^ Department of Thoracic Surgery, Sun Yat-sen University Cancer Center, State Key Laboratory of Oncology in South China, Collaborative Innovation Center of Cancer Medicine, Guangzhou, China

**Keywords:** small pulmonary nodules, video-assisted thoracoscopic surgery, hookwire, localization, treatment

## Abstract

**Purpose:**

To assess the feasibility of computed tomography (CT)-guided localization using a specific long dual-barbed hookwire in resection of pulmonary nodules with the size of 20mm or less by video-assisted thoracoscopic surgery (VATS) and to discuss the necessity of early treatment of small pulmonary nodules by VATS.

**Results:**

All the nodules were successfully localized with hook wire under CT guidance. The 34 nodules had a mean diameter of 8.9 ± 3.8 mm and a mean distance from the most superficial edge of the nodules to the visceral pleura of 21.4 ± 12.4 mm. The mean length of time for CT-guided percutaneous localization was 9.0 ± 3.8 minutes. Asymptomatic pneumothorax and parenchyma hemorrhage were observed in 1 patient (3.2%) and 5 patients (16.1%), respectively. VATS successfully resected all the lesions after localization. The mean VATS operation time was 2.6 ± 1.2 hours (range, 0.8−5.2 hours). 24 (70.6%) malignant nodules and 10 benign nodules were discovered after surgery.

**Materials and Methods:**

Between March 2012 and August 2014, 31 patients with 34 small pulmonary lesions underwent CT-guided hook wire localization and VATS resection. The efficacy of preoperative localization was evaluated in terms of procedure time, VATS success rate and associated complications of localization. And the pathology and imaging diagnosis of all nodules were recorded.

**Conclusions:**

The CT-guided Hook-wire localization for pulmonary nodules with the size of 20 mm or less is an effective and safe technique to assist VATS. Once small pulmonary nodules change in size or number, it is necessary to treat in an early and aggressive way with minimally invasive surgery.

## INTRODUCTION

Recently, an increase in small pulmonary nodules (SPNs) has been identified by high resolution Computed Tomography (CT) in routine clinic, particularly in low dose CT screening of lung cancer for high-risk population [1]. However, with CT criteria [2], it is hard to confidently differentiate small malignant pulmonary nodules from benign ones. Therefore, histopathological diagnoses become the vital step in the management of pulmonary nodules. A transthoracic fine-needle biopsy may be considered, but the reported diagnostic yield is rather low [3]. With the development of thoracic surgery in the past 15 years, video-assisted thoracoscopic surgery (VATS) technique provides a minimally invasive strategy for diagnostic or therapeutic excision of SPNs [4, 5], but VATS is of limited value for pulmonary nodules that are too small or too far away from the visceral pleura to be palpable or detectable by thoracosopy. Failure to see or to palpate a SPN may lead to an increase in the conversion thoracotomy rate to 46% [6, 7]. Preoperative localization of those small impalpable lesions becomes necessary and important for accurate VATS procedure [8, 9]. Here we report our recent prospective study on early treatment of small pulmonary nodules with the size of 20mm or less by VATS combined with preoperative CT-guided localization using a specific long dual-barbed hookwire.

## RESULTS

There were a total of 34 pulmonary lesions with diameters of 20mm or less in the 31 patients selected, (11 males and 20 females) with the mean age of 52.5 ± 11.1 years (range, 28–78 years old). 26 patients had a single nodule and 3 patients had two nodules in the same lung. 2 patients had multiple nodules in bilateral lungs, but we chose the largest one in the peripheral pulmonary parenchyma for wedge resection and diagnose. The 34 SPNs had a mean maximal long-axis diameter of 8.9 ± 3.8 mm (range, 3–18 mm), and a mean distance from the most superficial edge of the nodules to the visceral pleura of 21.4 ± 12.4 mm (range, 5–50 mm). While 7 lesions (20.6%) appeared to be ground glass nodule (GGN), 27 nodules (79.4%) displayed to be solid nodule in peripheral lung. The imaging diagnosis of 7 GGNs: 3 indeterminate lesions, 1 benign lesion and 3 malignant lesions were included. The imaging diagnosis of 27 solid nodules: 9 indeterminate nodules, 14 malignant nodules and 4 benign nodules were included. The characteristics of 34 SPNs in 31 patients were shown in Table 1.

**Table 1:** The characteristics of 34 small pulmonary nodules in 31 patients

Patient No.	Gender	Age	Diameter (mm)	Distance to Pleural (mm)	Imaging characteristic	Image Diagnosis	Localization Time (min)	Pathological Result
**1**	F	45	5	22	solid nodule	malignant	13	metastatic endometrial cancer
**2**	F	55	6	24	solid nodule	indeterminate	8	focal necrosis with calcification
**3**	F	59	10	32	GGN	malignant	7	carcinoma *in situ*
**4**	F	58	10	19	GGN	indeterminate	7	inflammatory changes
**5**	M	69	9	46	solid nodule	indeterminate	6	moderately to poorly differentiated adenocarcinoma
**6**	F	40	3	9	solid nodule	indeterminate	8	atypical adenocarcinoma
			5	14	solid nodule		7	atypical adenocarcinoma
**7**	M	62	8	39	GGN	malignant	23	metastatic lung adenocarcinoma
**8**	M	60	18	43	solid nodule	malignant	5	tuberculosis
**9**	M	58	8	5	solid nodule	malignant	7	metastatic lung adenocarcinoma
**10**	F	39	10	5	solid nodule	malignant	5	moderately differentiated adenocarcinoma
**11**	M	53	7	16	solid nodule	indeterminate	9	alveolar epithelial cell hyperplasia
**12**	F	28	9	12	solid nodule	indeterminate	9	carcinoma *in situ*
**13**	F	37	10	35	solid nodule	benign	5	tuberculosis
**14**	M	48	6	16	solid nodule	malignant	7	metastatic colon cancer
**15**	F	53	8	51	solid nodule	indeterminate	10	inflammatory changes
**16**	F	63	9	22	solid nodule	malignant	8	moderately differentiated adenocarcinoma
**17**	M	50	12	21	solid nodule	malignant	11	well to moderately differentiated adenocarcinoma
			6	20	solid nodule	malignant	11	inflammatory changes
**18**	F	59	13	19	solid nodule	malignant	10	moderately differentiated adenocarcinoma
**19**	F	47	8	8	solid nodule	benign	3	lymph node
**20**	F	47	3	7	solid nodule	indeterminate	6	carcinoma *in situ*
**21**	F	54	8	34	solid nodule	malignant	13	metastatic colon cancer
**22**	F	65	17	9	solid nodule	malignant	13	moderately differentiated adenocarcinoma
**23**	F	37	6	18	solid nodule	benign	5	carcinoma *in situ*
**24**	F	65	9	20	solid nodule	malignant	12	inflammatory changes
**25**	F	51	16	13	GGN	malignant	14	moderately differentiated adenocarcinoma
**26**	M	51	9	39	GGN	indeterminate	7	well-differentiated adenocarcinoma
			3	13	solid nodule	indeterminate	10	inflammatory changes
**27**	M	78	13	29	solid nodule	malignant	6	well-differentiated mucinous carcinoma
**28**	F	45	9	18	GGN	indeterminate	9	carcinoma *in situ*
**29**	M	34	6	14	solid nodule	benign	9	metastatic thyroid papillary carcinoma
**30**	F	58	7	6	GGN	benign	14	carcinoma *in situ*
**31**	M	59	15	29	solid nodule	malignant	10	moderately to poorly differentiated adenocarcinoma
**Mean ± SD**	**-**	**52.5 ± 11.1**	**8.9 ± 3.8**	**21.4 ± 12.4**	**-**	**-**	**9.0 ± 3.8**	-

GGN: ground-glass nodule.

F: female.

M: male.

SD: standard deviation.

**Figure 1:**
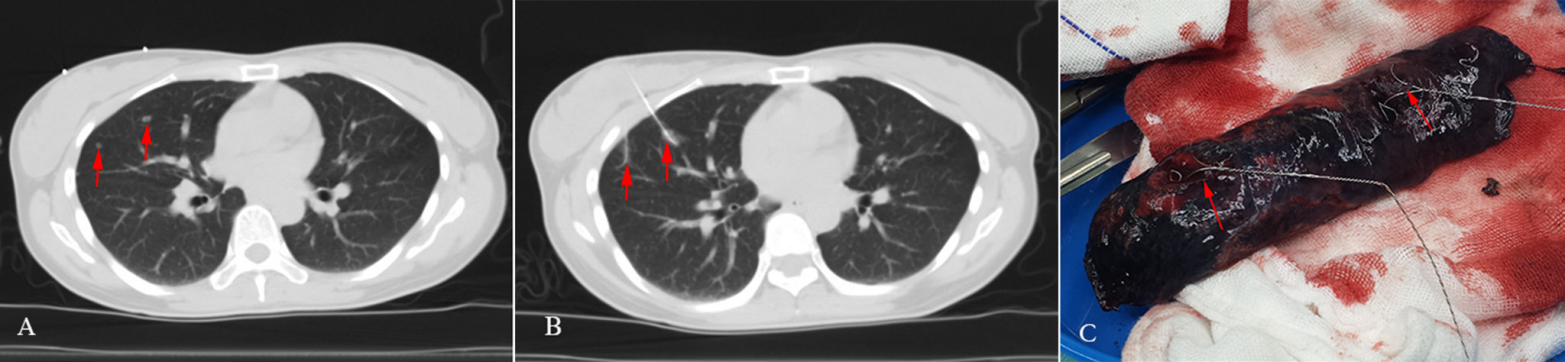
A 40-year-old female had two solid opacities with the diameter of 3 mm and 5 mm in the right middle lobe, respectively; the preoperative imaging diagnoses were considered to be indeterminate The two patients then received the right middle lobe wedge resection, and the immediate frozen-resection histopathological results came out to be two atypical adenocarcinomas. There was no additional lobectomy performed. (**A**) Chest transvers-resection enhanced CT scan shows: two solid lesions in the right middle lobe (red arrows); (**B**) Two hookwires are placed through the lesions under CT guidance (red arrows); (**C**) The hookwires (red arrows) insert through the SPNs in pathologic specimen.

All the 34 SPNs were successfully localized with 34 dual-barbed hookwires in the 31 patients. The mean length of time for CT-guided percutaneous localization was 9.0 ± 3.8 minutes (range, 3–23minutes), and the mean numbers of needle insertions or adjustments were 3.4 ± 1.4 times (range, 2–7times). Two hookwire placements were simultaneously performed in 3 patients with 2 target lesions (Figure 1). There was no need to re-locate in all cases. Asymptomatic pneumothorax and parenchyma hemorrhage were observed in 1 patient (3.2%) and 5 patients (16.1%), respectively.

All 34 nodules in the 31 patients were completely removed by VATS. The mean VATS operation time was 2.6 ± 1.2 hours (range, 0.8–5.2hours). Of the total 34 SPNs, there were 24 (70.6%) malignant nodules and 10 (29.4%) benign nodules. Histological results were diagnosed by experienced pathologists (as shown in Table 1). 14 Malignant lesions received additional lobectomy and mediatrinal lymph node cleaning-up had the following diagnoses: 9 adenocarcinomas, 1 well-differentiated mucinous carcinoma, and 4 carcinomas *in situ*. 10 benign nodules which underwent wedge resection, had the following diagnoses: 2 tuberculosis, 5 inflammatory nodules, 1 intrapulmonary lymph node, 1 focal necrosis with calcification, and 1 alveolar epithelial cell hyperplasia. There were 10 nodules of malignant diagnoses removed at wedge resection without additional lobectomy: 2 carcinomas *in situ*, 2 metastatic colon cancer, 1 metastatic endometrial cancer, 1 metastatic thyroid papillary carcinoma, 2 metastatic lung moderately differentiate adenocarcinoma, and 2 atypical adenocarcinoma. The 2 patients with carcinoma *in situ* who only received wedge resection due to the following reasons: the frozen histopathological result was undefined during the operation, the subsequently paraffin section histopathological result came out to be carcinoma *in situ*, but the patients refused the recommended further lobectomy and lymph node resection. Postoperative pneumothorax occurred in 3 (9.7%) cases, which were successfully managed by closed thoracic drainage. Subcutaneous emphysema occurred in 2 (6.5%) cases.

**Figure 2:**
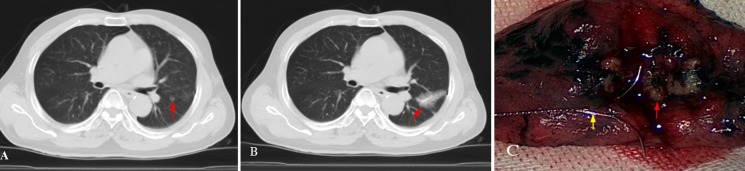
A 62-year-old male had a ground-glass nodule in the left upper lobe with the diameter of 8 mm on chest CT scan; the preoperative imaging diagnosis was considered to be indeterminate The patient received the left upper lobe wedge resection, and the immediate frozen-resection pathological result came out to be moderately differentiated adenocarcinoma. Additional lobectomy and lymph node cleaning-up were then performed. (**A**) Chest transverse-resection enhanced CT scan shows: a GGN in the left upper lobe (red arrow); (**B**) A hookwire is placed through the nodule successfully under CT guidance (red arrow). A small amount of pulmonary parenchyma hemorrhage appears during the localization procedure; (**C**) The hookwire (yellow arrow) inserts through the white SPN (red arrow) in pathologic specimen.

There were 27 solid lesions including 9 (33.3%) benign lesions and 18 (66.7%) malignant lesions (Figure 2). The proportions of malignant solid nodules with different diameters were shown in Table 2A. The preoperative imaging diagnoses of 13 (48.1%) solid nodules were consistent with the postoperative histopathological results, 5 (18.5%) solid nodules were misdiagnosed. And in the remaining 9 (33.3%) solid nodules with the preoperative imaging diagnoses of indetermination, 4 (44.4%) benign nodules and 5 (55.6%) malignant lesions were found by the postoperative histopathological examination. Meanwhile, there were 7 grand-glass nodules, including 1 (14.3%) benign lesions and 6 (85.7%) malignant lesions (Figure 2). The portions of malignant GGNs with different diameter were shown in Table 2B. The preoperative imaging diagnoses of 3 (42.9%) GGNs were consistent with the postoperative histopathological results, 1 (14.3%) GGN was misdiagnosed. Of the remaining 3 (42.9%) GGNs with the preoperative imaging diagnoses of indetermination, 1 (33.3%) benign nodules and 2 (66.7%) malignant lesions were found. The image diagnoses and pathologic results in different sizes of SPNs are shown in Table 1.

**Table 2:** Malignancy rates in different size of small pulmoary nodules

2A: Malignancy rates in different size of solid nodules			
Diameter (mm)	Number	Number of malignant nodules	Malignancy rate
≤ 5.0	5	4	80%
5.0–10.0	16	9	56.30%
10.0–15.0	4	4	100%
15.0–20.0	2	1	50%
Total	27	18	66.67%

2B: Malignancy rates in different size of ground-glass nodules			
Diameter (mm)	Number	Number of malignant nodules	Malignancy rate
≤ 5.0	–	–	–
5.0–10.0	6	5	83.33%
10.0–15.0	–	–	–
15.0–20.0	1	1	100%
Total	7	6	85.71%

## DISCUSSION

These SPNs present radiologists and clinicians with diagnostic and therapeutic challenges. With the development of modern medical imaging technology, these challenges are increasingly incisive and prominent. Lack of typical imaging features increases the difficulties in qualitative diagnosis of these SPNs. When dealing with SPNs, CT follow-up is the most commonly recommended and used method [10]. However, if the SPN is malignant, patients often miss the best treatment time, leading to tumor progression, and consequently a shortened life.

Among all the 34 SPNs in this study, malignant lesions accounted for 70.6% (*n* = 24). As to the 27 solid lesions and the 7 GGNs, malignant nodules made up for 66.7% and 85.7%, respectively. These data illustrate the fact that these SPNs with the diameter of 20 mm or less have a high rate of malignancy. What's more, there were 9 indeterminate lesions and 5 misdiagnosed lesions among the 27 solid nodules. In the 9 indeterminate lesions, malignant nodules accounted for 55.6% (5/9). Of the 5 misdiagnosed solid nodules, 2 nodules, which had the imaging diagnoses of benign lesions, turned out to be malignant. Meanwhile, there were 3 indeterminate lesions and 1 misdiagnosed lesions among the 7 GGNs. In the 3 indeterminate GGNs, malignant nodules accounted for 66.7% (2/3). There was one misdiagnosed GGN, which had the imaging diagnoses of benign lesion, turned out to be malignant. As a result, it is urgency for us to make early and effective diagnoses and treat these SPNs with the diameter of 20 mm or less. Closely follow-up observation is recommended. And Once the small pulmonary nodule changes in size or number, it is necessary to treat SPNs in an early and aggressive way with minimally invasive surgery.

**Figure 3:**
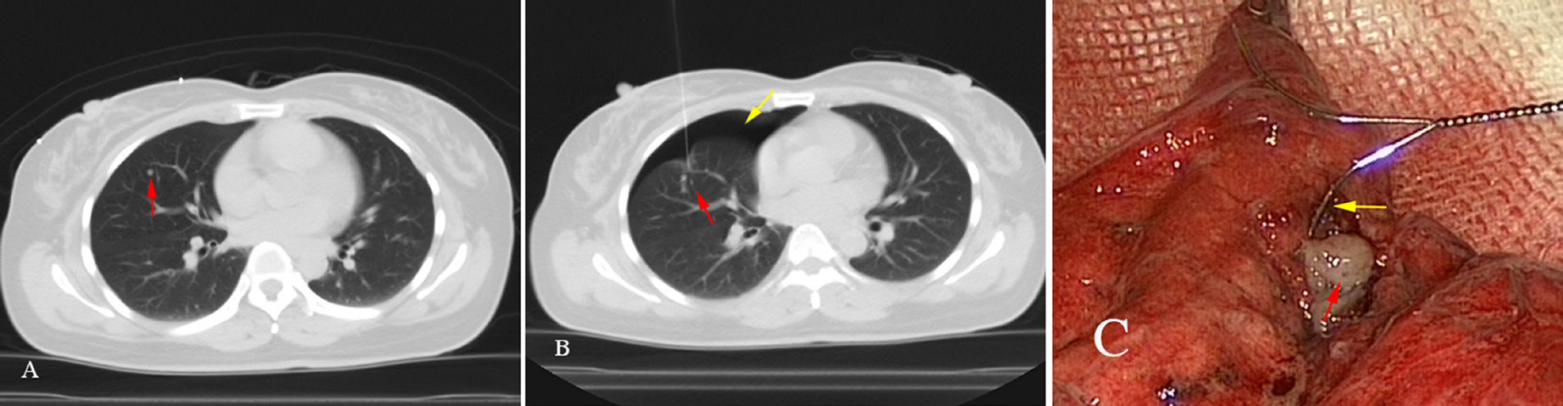
A 45-year-old female with a history of endometrial cancer for 2 years, had a solid nodule of 4 mm in the right middle lobe on chest CT scan During the 3 months follow-ups, there was no change in size. It was difficult to distinguish whether the SPN was malignancy or not, leading to unable to determine the tumor staging. The doctor suggested to continue follow-up observations, but patient screamed for surgical treatment. The patient then received the right middle lobe wedge resection. The immediate frozen-section histopathological result turned out to be a moderately differentiated adenocarcinoma, considering the endometrial adenocarcinoma of lung metastasis. The clinical staging came out to be stage IV. (**A**) Chest transvers-resection enhanced CT scan shows: a solid nodule in the right middle lobe (red arrow); (**B**) A hookwire is placed through the nodule successfully under CT guidance (red arrow). Pneumothorax appears during the localization procedure (yellow arrow); (**C**) The hookwire (yellow arrow) inserts through the white SPN (red arrow) in pathologic specimen.

As to the patients who had SPNs and primary tumors in other parts of the body simultaneously or with a history of malignant tumor, the clinical staging and treatment protocols were directly determined according to the pathological characteristics of SPNs. In our study, two patients (Patient No.2 and No.13) had SPNs and primary tumors in other parts of body at the same time. These two female patients both have cervical cancer as the primary cancer and a SPN simultaneously, and both received lobe wedge resection. The histopathological results were focal necrosis with calcification and tuberculosis, respectively. The clinical staging came out to be stage IB1 other than stage IV. These patients then received laparoscopic cervical cancer radical excision, sequentially. No further treatment was needed after surgery. Meanwhile, there were 6 patients (Patient No.1, 14, 21, 9, 25 and 6) with SPNs having a history of malignant tumor. The primary tumors had all been removed surgically. For the first 4 patients, the SPNs were all diagnosed to be metastatic after lobe wedge resection (Figure 3). The tumor stage came out to be stage IV. And then they all received systemic chemotherapy. As to the fifth patient, Wedge resection was conducted firstly. Additional lobe resection and lymph node cleaning-up were then performed with immediate frozen histopathological result of moderately differentiated adenocarcinoma. The disease was diagnosed to be stage I non-small cell lung cancer (NSCLC) instead of metastatic tumor. Moreover, the remaining patient (Patient No.6) with a history of malignant mesothelioma for 4 years had two solid nodules in the right middle lobe. The wedge resection was conducted, and the histopathological results turned out to be atypical adenocarcinoma, which was difficult to distinguish between primary tumors and metastatic tumors, but could rule out metastases from malignant mesothelioma. Further examinations were needed. Notably, there was one patient (Patient No.29) with multiple SPNs in both lungs received a preoperative image diagnosis of acute miliary tuberculosis, but the patient failed to respond to the anti-tuberculosis treatment. Wedge resection was conducted to get a definite diagnosis. And the histopathological result came out to be metastatic thyroid papillary carcinoma. According to the histopathological result, a further thyroid ultrasound examination was conducted after VATS, and multiple nodules were detected in bilateral thyroid. The patients then received thyroidectomy with the postoperative histopathological result of thyroid papillary carcinoma. The pathological characteristics of SPNs provided precise clues for seeking the primary tumors. Therefore, early and aggressively therapy of SPNs is of great importance in diagnosis and treatment of diseases, especially for the patients who had SPNs and primary tumors in other parts of the body simultaneously, or with a history of malignant tumor.

High-resolution CT technique allows more SPNs to be detected [8]. These SPNs requires bronchoscopy, TTNB or even surgery for insured diagnosis [11]. However, a meta-analysis carried out by Wang Memoli JS et al. has shown: the diagnostic rate of different bronchoscopy biopsy techniques in SPNs with the diameter of 20mm or less is only 61% (54%–68%) [12]. As to TTNB, Gould MK et al. reported that the diagnostic rate of SPNs less than 15mm is only 70%–80% [13]. VATS resection provides adequate tissue for histopathological examination, making accurate pathological diagnosis for all these SPNs. Comparing with the conventional surgery, VATS has the following advantages: a more reasonable operation resection scope, minimal invasion, short operation time, and less postoperative complications [14]. Additionally, a meta-analysis conducted by Fan J et al. shows: for SPNs with the diameter of 20 mm or less, sub-segment resection (including wedge resection) has similar long-term survival rate as lobectomy in stage I NSCLC patients [15]. A Japanese multicenter randomized prospective study suggests that the sub-segment resection can replace lobectomy in T1N0M0 NSCLCs, especially for SPNs that are smaller than 20 mm [16]. In our study, pneumothorax occurred in 3 (9.7%) cases, but they were successfully managed by closed thoracic drainage. VATS provides a solid technical support and theoretical basis for the early treatment of SPNs.

However, VATS is limited to the pulmonary nodules that are too small or too far away from the pleural surface to be visualized by thoracosopy or to be palpable. Failure to detect a lesion may lead to an increase in the conversion thoracotomy rate to 46% [7, 8]. CT-guided localization is fundamental to ensure accurate resection of these SPNs. In our study, all 34 SPNs were successfully localized and resected. Asymptomatic pneumothorax and parenchyma hemorrhage were observed in 1 (3.2%) and 5 (16.1%) during the localization procedures, which was a little lower than those Yeow KM et al. had reported [17]. The mean length of time for CT-guided percutaneous localization was 9.0 ± 3.8 minutes (range, 3–23 minutes), and the mean number of needle insertions or adjustments was 3.4 ± 1.4 times (range, 2–7times). The procedure of CT-guided localization with the dual-barbed wire was relatively quick and safe. Compared with the reported 54% successful resection rate of the non-guided VATS, the union of CT-guided localization with the dual-barbed wire and VATS has a macro improvement in resection rate [18]. CT-guided localization provides VATS with precise positioning, makes the lung nodules which are too small or distant from the pleural surface to be resectable by VATS, ensures the complete resection of pulmonary nodules within the maximum retention of normal lung tissue, and reduces the rate of thoracotomy effectively [6–7].

Regarding the best way to locate a subcentimetre SPN to achieve successful excision for patients undergoing BATS excision, several techniques for SPN localization have been reported [8], including finger palpation [19], methylene blue dye [20], contrast medium [21], ultrasonography [22], radio-Tc99 [23], microcoil [18], spiral-wire [24] and single-barbed hook-wire [25], but each has limitations that may prevent it from being widely adopted in clinic practice. Among these approaches, the hook-wire with one barb was potentiated to be useful for its low complication rate and minimal trauma [26, 27], but its dislodgement rate was rather high as reported [28, 29]. We had tried 3 cases with single-barbed hook-wire for CT-guided localization before, but dislodgement occurred in 2 cases. The hook-wire with double barbs provides more anchoring power for traction on pulmonary nodules during VATS, and the marked nodule can be pulled towards the outside of the lung and held for VATS, allowing the exact placement of the linear endostapler for high effective wedge excision. Notably, there was one dislodgement happened. In this case, the distance from the most superficial edge of the nodule to the visceral pleural was only 5mm leading to a shallow localization. The hookwire was prolapsed from the collapsing lung tissue during the VATS procedure, however the nodule was successfully resected using the traces, which include small hemorrhage and scarification on the parietal pleura caused by the dual-barbed wire.

Our study had several limitations. All the 34 SPNs were resected at only one medical center. Sample size was relatively small in our study. We are planning to conduct a randomized prospective study with large sample size for further investigations.

## MATERIALS AND METHODS

### Patient selection

From March 2012 to August 2014, patients with SPNs ≤ 20 mm were selected for a combination study using both CT-guided dual-barbed hookwire localization and VATS. The inclusion criteria: the small pulmonary nodule stayed no change since founded in patients without tumor-related diseases, but the size or the number of the SPN increased recently; the small pulmonary nodule stayed no change since founded, but the patients strongly required surgical treatment; a or several small pulmonary nodules were detected in patients who had a history of tumor-related disease or tumor simultaneously. Written informed consent was obtained from all the patients after explaining the necessity and risks. VATS was recommended for further diagnosis and/or treatment of the detected lesions. In all cases, three interventional radiologists and one surgeon reviewed all the available CT scans. The institutional review board approved the protocol. Demographic data, clinical, biological and morphological findings, treatments, and outcomes were recorded.

### CT Scan and needle preparation

**Figure 4:**
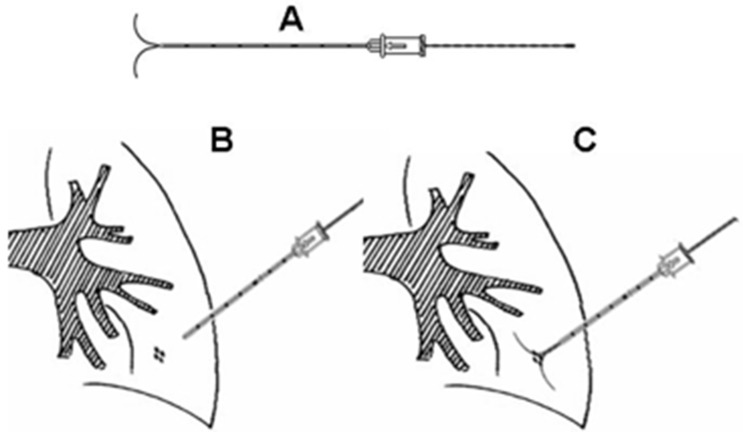
The long dual-barbed hookwire and its placement scheme that we utilized in current study (**A**) The long hookwire with two barbs; (**B**) The hookwire was placed through the lesion in the lung; (**C**) The barbs were released to anchor, and trace the lesion for VATS.

A multidetector CT helical scanner (Phillips Brilliance Big Bore, Philips Medical Systems Technologies LTD) was used, with the simultaneous acquisition of 16 slices per full rotation. The technical parameters were: 120 kV, 250 mAs, collimation 6 × 2 mm, slice 1 mm, reconstruction increment 1 mm. The dual-barbed wire system (breast lesion localization wire) consisted of a 20-gauge, 10.7 cm long, calibrated cannula and a 20 cm long calibrated wire with two thorns (Bard Dualok, Bard Peripheral Vascular, Inc.). The cannula had stenciled marks every 10 mm on the outside of the needle shaft. (Figure 4).

### CT-guided percutaneous localization

With the previously available CT scans for guidance, patient was appropriately positioned, and improvised metal markers were used to determine if the puncture site was properly placed. CT scan was then performed to identify the suspicious nodule. Routine breathing instructions were not given during preliminary imaging or during localization procedure unless deemed necessary by the treating physician. An optimal access route was designed by measuring the distance from the skin to the superficial edge of SPNs and choosing an entry point. After disinfection of the skin and a subcutaneously local anesthesia (1% lidocaine hydrochloride) around the marked area, the cannula needle with the dual-barbed wire was gradually inserted through the pulmonary parenchyma into the nodule. While the outer cannula needle was withdrawn, the dual barb could be released. A postprocedure CT scan was performed to confirm the final position of the dual-barbed wire, and to assess for any existing complications. CT-guided localization was scheduled 2 hours before VATS surgery. The period of time patients spent from receiving a subcutaneously local anesthesia to the withdrawing of the outer cannula needle was regarded as the localization procedure time.

### Video-assisted thoracoscopy surgery (VATS)

VATS was performed under general anesthesia using single lung ventilation via a double-lumen endobronchial tube. In most cases, the thoracoscope was inserted through a 11.5 mm thoracic port in the seventh intercostal space on the midaxillary line. The procedure involved making another 11.5 mm thoracic port for the endoscopic stapler, and a 5.5 mm thoracic port for the lung forceps. The wire was easily pulled for tracing SPN in the pleural cavity without dislodging the hook-head when the lung collapsed. The lesion was sequentially resected. The resected wire and lung tissue were packed into surgical bags to prevent metastatic implantation of malignant disease and were withdrawn from the chest via an intercostal incision. All the resected lung specimens were immediately sent for frozen-section histopathological examination.

### Immediate frozen histopathological examination

A frozen-section specimen was removed from the pulmonary nodules for histopathological diagnosis. If the nodule was diagnosed to be benign or metastatic, no further resection was performed. The pleural cavity was douched with saline solution, and then a chest tube with underwater drainage system was placed. When the nodule was diagnosed to be primary lung cancer, VATS lobectomy and mediastinal lymph node cleaning-up were performed.

### Statistical analyses

SPSS for OS X, version 20.0 (SPSS, Chicago, III), was used to access all the statistical analyses. Data were presented as mean ± standard deviation (SD), unless otherwise indicated.

## CONCLUSIONS

In summary, the combination of CT-guided localization using a dual-barbed hookwire with VATS is a safe, convenient and efficient technique for early treatment of SPN that should be widely expanded in clinical practice. And we should draw our attention to the small pulmonary nodule with the diameter of 20 mm or less, and closely follow-up observation is recommended. Once the small pulmonary nodule changes in size or number, it is necessary to treat SPNs in an early and aggressive way with minimally invasive surgery.

## Abbreviations

VATS: video-assisted thoracoscopic surgery
CT: computed tomography
SPN: small pulmonary nodule
GGN: ground glass nodule
TTNB: transthoracic needle biopsy

## References

[1] Swensen SJ, Jett JR, Hartman TE, Midthun DE, Mandrekar SJ, Hillman SL, Sykes AM, Aughenbaugh GL, Bungum AO, Allen KL (2005). CT screening for lung cancer: five-year prospective experience. Radiology.

[2] Leef JL, Klein JS (2002). The solitary pulmonary nodule. Radiol Clin North Am.

[3] Kothary N, Lock L, Sze DY, Hofmann LV (2009). Computed tomography-guided percutaneous needle biopsy of pulmonary nodules: impact of nodule size on diagnostic accuracy. Clin Lung Cancer.

[4] Lewis RJ, Caccavale RJ, Sisler GE (1992). Imaged thoracoscopic lung biopsy. Chest.

[5] Hirai S, Hamanaka Y, Mitsui N, Morifuji K, Uegami S (2006). Role of video-assisted thoracic surgery for the diagnosis of indeterminate pulmonary nodule. Ann Thorac Cardiovasc Surg.

[6] Yeh YC, Kadota K, Nitadori J, Sima CS, Rizk NP, Jones DR, Travis WD, Adusumilli PS (2016). International Association for the Study of Lung Cancer/American Thoracic Society/European Respiratory Society classification predicts occult lymph node metastasis in clinically mediastinal node-negative lung adenocarcinoma. Eur J Cardiothorac Surg.

[7] Pittet O, Christodoulou M, Pezzetta E, Schmidt S, Schnyder P, Ris HB (2007). Video-assisted thoracoscopic resection of a small pulmonary nodule after computed tomography guided localization with a hook-wire system. Experience in 45 consecutive patients. World J Surg.

[8] Zaman M, Bilal H, Woo CY, Tang A (2012). In patients undergoing video-assisted thoracoscopic surgery excision, what is the best way to locate a subcentimetre solitary pulmonary nodule in order to achieve successful excision?. Interact Cardiovasc Thorac Surg.

[9] Chen S, Zhou J, Zhang J, Hu H, Luo X, Zhang Y, Chen H (2011). Video-assisted thoracoscopic solitary pulmonary nodule resection after CT-guided hookwire localization: 43 cases report and literature review. Surg Endosc.

[10] Kobayashi Y, Fukui T, Ito S, Usami N, Hatooka S, Yatabe Y, Mitsudomi T (2013). How long should small lung lesions of ground-glass opacity be followed?. J Thorac Oncol.

[11] Das-Neves-Pereira JC, Bagan P, Coimbra-Israel AP, Grimaillof-Junior A, Cesar-Lopez G, Milanez-de-Campos JR, Riquet M, Biscegli-Jatene F (2009). Fast-track rehabilitation for lung cancer lobectomy: a five-year experience. Eur J Cardiothorac Surg.

[12] Wang Memoli JS, Nietert PJ, Silvestri GA (2012). Meta-analysis of guided bronchoscopy for the evaluation of the pulmonary nodule. Chest.

[13] Liu CY, Lin CS, Shih CH, Liu CC (2014). Single-port video-assisted thoracoscopic surgery for lung cancer. J Thorac Dis.

[14] Fan J, Wang L, Jiang GN, Gao W (2012). Sublobectomy versus lobectomy for stage I non-small-cell lung cancer, a meta-analysis of published studies. Ann Surg Oncol.

[15] Okada M, Koike T, Higashiyama M, Yamato Y, Kodama K, Tsubota N (2006). Radical sublobar resection for small-sized non-small cell lung cancer: a multicenter study. J Thorac Cardiovasc Surg.

[16] Yeow KM, See LC, Lui KW, Lin MC, Tsao TC, Ng KF, Liu HP (2001). Risk factors for pneumothorax and bleeding after CT-guided percutaneous coaxial cutting needle biopsy of lung lesions. J Vasc Interv Radiol.

[17] Suzuki K, Nagai K, Yoshida J, Ohmatsu H, Takahashi K, Nishimura M, Nishiwaki Y (1999). Videoassisted thoracoscopic surgery for small indeterminate pulmonary nodules: indications for preoperative marking. Chest.

[18] Gonfiotti A, Davini F, Vaggelli L, De Francisci A, Caldarella A, Gigli PM, Janni A (2007). Thoracoscopic localization techniques for patients with solitary pulmonary nodule: hookwire versus radio-guided surgery. Eur J Cardiothorac Surg.

[19] Lenglinger FX, Schwarz CD, Artmann W (1994). Localization of pulmonary nodules before thoracoscopic surgery: value of percutaneous staining with methylene blue. AJR Am J Roentgenol.

[20] Choi BG, Kim HH, Kim BS, Kim KT, Shinn KS, Moon SW (1998). Pulmonary nodules: CT-guided contrast material localization for thoracoscopic resection. Radiology.

[21] Greenfield AL, Steiner RM, Liu JB, Cohn HE, Goldberg BB, Rawool NM, Merton DA (1997). Sonographic guidance for the localization of peripheral pulmonary nodules during thoracoscopy. AJR Am J Roentgenol.

[22] Chella A, Lucchi M, Ambrogi MC, Menconi G, Melfi FM, Gonfiotti A, Boni G, Angeletti CA (2000). A pilot study of the role of TC-99 radionuclide in localization of pulmonary nodular lesions for thoracoscopic resection. Eur J Cardiothorac Surg.

[23] Eichfeld U, Dietrich A, Ott R, Kloeppel R (2005). Video-assisted thoracoscopic surgery for pulmonary nodules after computed tomography-guided marking with a spiral wire. Ann Thorac Surg.

[24] Shah RM, Spirn PW, Salazar AM, Steiner RM, Cohn HE, Solit RW, Wechsler RJ, Erdman S (1993). Localization of peripheral pulmonary nodules for thoracoscopic excision: value of CT-guided wire placement. AJR Am J Roentgenol.

[25] Thaete FL, Peterson MS, Plunkett MB, Ferson PF, Keenan RJ, Landreneau RJ (1999). Computed tomography-guided wire localization of pulmonary lesions before thoracoscopic resection: results in 101 cases. J Thorac Imaging.

[26] Miyoshi K, Toyooka S, Gobara H, Oto T, Mimura H, Sano Y, Kanazawa S, Date H (2009). Clinical outcomes of short hook wire and suture marking system in thoracoscopic resection for pulmonary nodules. Eur J Cardiothorac Surg.

[27] Chen YR, Yeow KM, Lee JY, Su IH, Chu SY, Lee CH, Cheung YC, Liu HP (2007). CT-guided hook wire localization of subpleural lung lesions for video-assisted thoracoscopic surgery (VATS). J Formos Med Assoc.

[28] Ciriaco P, Negri G, Puglisi A, Nicoletti R, Del Maschio A, Zannini P (2004). Video-assisted thoracoscopic surgery for pulmonary nodules: rationale for preoperative computed tomography-guided hookwire localization. Eur J Cardiothorac Surg.

[29] Dendo S, Kanazawa S, Ando A, Hyodo T, Kouno Y, Yasui K, Mimura H, Akaki S, Kuroda M, Shimizu N, Hiraki Y (2002). Preoperative localization of small pulmonary lesions with a short hook wire and suture system: experience with 168 procedures. Radiology.

